# Black and African American Connections to Parkinson's Disease Study: Addressing Missing Diversity in Parkinson's Disease Genetics

**DOI:** 10.1002/mds.29042

**Published:** 2022-04-30

**Authors:** Sara Bandres‐Ciga, Black and African American Connections to Parkinson's Disease (BLAAC PD) Study Group

**Affiliations:** ^1^ Laboratory of Neurogenetics National Institute on Aging, National Institutes of Health Bethesda Maryland USA

Our current understanding of Parkinson's disease and atypical parkinsonism‐related syndromes is disproportionately based on studying populations of European ancestry, leading to a significant gap of knowledge concerning clinical features, genetics, and pathophysiology underlying disease etiology in underrepresented populations, including Black and African American individuals.

To date, an increasing number of common susceptibility loci for Parkinson's disease, along with rare and deleterious genetic variants responsible for monogenic cases,^1^, ^2^ are well established in populations of European, Latino and Asian ancestry.^3^, ^4^, ^5^ Nevertheless, the impact of both causal and risk genetic factors on Parkinson's disease in Black and African American individuals remains largely unknown. Notably, our preliminary scientific observations based on genetic assessments show significantly different distributions of cumulative genetic risk in the Black American and African American population in comparison with the European population when applying a genetic risk score composed of the 90 risk loci previously linked to European populations (Fig. 1).

**FIG 1:**
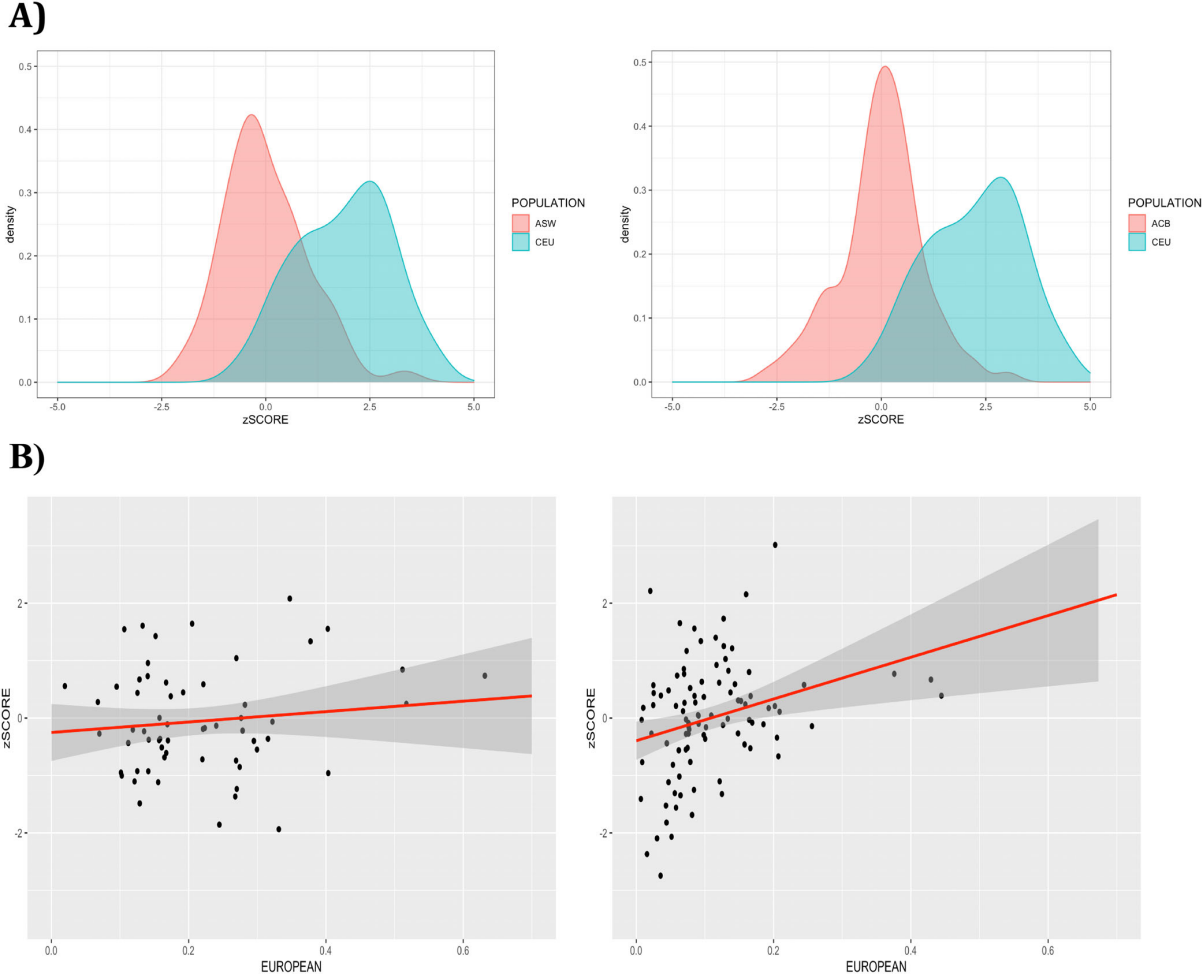
Parkinson's disease genetic risk score comparison based on known risk loci in European versus Black and African American populations. (**A**) Genetic risk score (GRS) including the 90 independent Parkinson's disease risk loci identified in European populations^3^ using publicly available whole‐genome sequencing data on GRCh38 format from the 1000 Genomes Project (https://www.internationalgenome.org/) for Europeans (CEU highlighted in blue) versus Black and African American populations of Southwest origin (ASW highlighted in red) and Europeans versus Afro‐Caribbeans (ACB highlighted in red). (**B**) Linear regression analyses of GRS versus percentage European ancestry predicting increased genetic risk. This model shows a positive association between increased European ancestry and increased genetic risk burden from the GRS; GRS versus % European in ASW ancestry populations: (beta = 0.023 ± 0.80; *p* = 0.004); GRS versus % European in ACB ancestry populations: (beta = 0.036 ± 1.24; *p* = 0.004). [Color figure can be viewed at wileyonlinelibrary.com]

As part of our commitment to address the lack of representation in genetics research, the Global Parkinson's Genetics Program (GP2)^6^ (https://gp2.org/), supported by the Aligning Science Across Parkinson's initiative^7^ and in collaboration with The Michael J. Fox Foundation for Parkinson's Research, has recently launched the Black and African American Connections to Parkinson's Disease (BLAAC PD) study (https://blaacpd.org). These pioneering efforts seek to change the landscape of our field by deciphering the genetic architecture of Parkinson's disease in traditionally underserved populations, maximizing both genetic discovery and improving the generalizability of research findings.

To further explore these differences, the BLAAC PD study will recruit Black and African American people with Parkinson's disease, as well as healthy subjects, from across the United States. As part of the pilot phase, BLAAC PD launched four sites that are actively recruiting participants: University of Chicago, Rush University, Kaiser Permanente Mid‐Atlantic States, and University of Alabama at Birmingham. BLAAC PD is now seeking to expand in terms of number of sites and infrastructure and aims to recruit 2000 cases and 2000 control subjects. This research project will generate valuable data that may ultimately reduce existing health inequities across race, ancestry, and geographical region. It is likely that population‐specific genomic variation may contribute to altered susceptibility and clinical manifestations of Parkinson's disease in Black and African American individuals. In addition, fine‐mapping analyses across this population could be extremely useful to provide insights applicable to a broad disease landscape, given the potential to identify putative functional variants.

BLAAC PD will integrate into the broad GP2 initiative, accelerating biological insight and identification of potential drug targets. This pioneering research project will give rise to the generation of critical data in a continuous effort to uncover the molecular complexity underlying Parkinson's disease etiology.

## Author Roles

Members of the BLAAC PD study drafted and made critical revisions to this article.

## Appendix

Members of the BLAAC PD Study Group are: Alyssa O'Grady (The Michael J. Fox Foundation for Parkinson's Research, New York, NY, USA); Andrew Singleton, PhD (Laboratory of Neurogenetics, National Institute on Aging, National Institutes of Health, Bethesda, MD, USA); Bernadette Siddiqi (The Michael J. Fox Foundation for Parkinson's Research, New York, NY, USA); Cabell Jonas, PhD (Mid‐Atlantic Permanente Research Institute [MAPRI], Rockville, MD, USA); Charisse Comart (The Michael J. Fox Foundation for Parkinson's Research, New York, NY, USA); Cornelis Blauwendraat, PhD (Laboratory of Neurogenetics, National Institute on Aging, National Institutes of Health, Bethesda, MD, USA); David Standaert, MD, PhD (Department of Neurology, University of Alabama at Birmingham, Birmingham, AL, USA); Deborah Hall, MD, PhD (Department of Neurological Sciences, Rush University Medical Center, Chicago, IL, USA); Divya Menghani (Department of Neurology, University of Chicago Medicine, Chicago, IL, USA); Ejaz A. Shamin, MD (Mid‐Atlantic Permanente Research Institute [MAPRI], Rockville, MD, USA); Jared Williamson (Mid‐Atlantic Permanente Research Institute [MAPRI], Rockville, MD, USA); Joseph Richardson (Department of Neurology, University of Alabama at Birmingham, Birmingham, AL, USA); Justin C. Solle, MBA (The Michael J. Fox Foundation for Parkinson's Research, New York, NY, USA); Mike A. Nalls, PhD (Laboratory of Neurogenetics, National Institute on Aging, National Institutes of Health, Bethesda, MD, USA); Mahesh Padmanaban, MD (Department of Neurology, University of Chicago Medicine, Chicago, IL, USA); Maggie Kuhl (The Michael J. Fox Foundation for Parkinson's Research, New York, NY, USA); Marc Rosenbaum (Department of Neurological Sciences, Rush University Medical Center, Chicago, IL, USA); Marissa Dean, MD (Department of Neurology, University of Alabama at Birmingham, Birmingham, AL, USA); Sara Bandres‐Ciga, PharmD, PhD (Laboratory of Neurogenetics, National Institute on Aging, National Institutes of Health, Bethesda, MD, USA); and Tao Xie, MD (Department of Neurology, University of Chicago Medicine, Chicago, IL, USA).
